# Prevalence of Chronic Conditions in Australia

**DOI:** 10.1371/journal.pone.0067494

**Published:** 2013-07-23

**Authors:** Christopher Harrison, Helena Britt, Graeme Miller, Joan Henderson

**Affiliations:** 1 Family Medicine Research Centre, Sydney School of Public Health, University of Sydney, Parramatta, NSW, Australia; Zhongshan Ophthalmic Center, China

## Abstract

**Objectives:**

To estimate prevalence of chronic conditions among patients seeing a general practitioner (GP), patients attending general practice at least once in a year, and the Australian population.

**Design, setting and participants:**

A sub-study of the BEACH (Bettering the Evaluation and Care of Health) program, a continuous national study of general practice activity conducted between July 2008 and May 2009. Each of 290 GPs provided data for about 30 consecutive patients (total 8,707) indicating diagnosed chronic conditions, using their knowledge of the patient, patient self-report, and patient's health record.

**Main outcome measures:**

Estimates of prevalence of chronic conditions among patients surveyed, adjusted prevalence in patients who attended general practice at least once that year, and national population prevalence.

**Results:**

Two-thirds (66.3%) of patients surveyed had at least one chronic condition: most prevalent being hypertension (26.6%), hyperlipidaemia (18.5%), osteoarthritis (17.8%), depression (13.7%), gastro-oesophageal reflux disease (11.6%), asthma (9.5%) and Type 2 diabetes (8.3%). For patients who attended general practice at least once, we estimated 58.8% had at least one chronic condition. After further adjustment we estimated 50.8% of the Australian population had at least one chronic condition: hypertension (17.4%), hyperlipidaemia (12.7%), osteoarthritis (11.1%), depression (10.5%) and asthma (8.0%) being most prevalent.

**Conclusions:**

This study used GPs to gather information from their knowledge, the patient, and health records, to provide prevalence estimates that overcome weaknesses of studies using patient self-report or health record audit alone. Our results facilitate examination of primary care resource use in management of chronic conditions and measurement of prevalence of multimorbidity in Australia.

## Introduction

The ageing of the population [1] is expected to lead to increases in prevalence of chronic conditions, multimorbidity [2], and the demand on primary care [3]. To enable the health systems to respond to these increases, the prevalence of chronic conditions needs to be measured in an accurate and timely manner. There are three major methods by which prevalence is usually measured: respondent self-report; health record audit; and screening.

Many governments use large population health surveys that rely on respondent self-report to measure the prevalence of chronic conditions [4]–[6]. One such study is the National Health Survey [7] (NHS), one of Australia's largest studies of chronic conditions, which relies primarily on respondent self-report despite well documented concerns about the validity and reliability of self-reported health information [8]–[12].

Using health records (paper and/or electronic) to estimate prevalence is often seen as superior to patient self-report [13]–[15]. However, the quality of information in health records can be compromised through inaccurate [16]–[18] or incomplete records [9], [15], and there are often issues in obtaining patient consent. Studies that screen the population, such as the Australian Diabetes, Obesity and Lifestyle Study (AusDiab) [19], avoid these issues, but are usually limited to a specific disease or groups of diseases and are relatively expensive - the most recent AusDiab study costing over $2.5 million [20].

Australia has a universal medical insurance scheme called Medicare which (fully or partially) covers the individuals cost of visits to general practitioners (GPs). GPs provide the bulk of primary care and act as gate keepers to government-subsidised health care from other medical specialists. The BEACH (Bettering the Evaluation And Care of Health) program is a study of general practice activity in Australia. Sub-studies of the BEACH program can provide national prevalence estimates for chronic conditions, free of the limitations of health record audits and patient self-report. Our earlier research [21] showed that by embedding sub-studies within the national BEACH program [22], we could gain timely, accurate prevalence estimates of common chronic conditions. Accuracy was achieved by using the GP as an expert interviewer and informant, drawing on their knowledge of the patient, the patient's knowledge and the patient's health record.

This paper builds on our earlier methods by expanding the study's scope to include all chronic conditions (rather than a selection of common chronic conditions) and by improving the method of dealing with non-attenders when estimating population prevalence. This paper will show that by utilising the GP as an expert interviewer within the existing BEACH infrastructure, we can overcome the limitations of patient self-report, or patient health record review alone, to estimate prevalence of chronic conditions in Australia, at a marginal cost to the overall BEACH program.

## Methods

In this study, patients attending a subsample of GPs participating in the BEACH program were surveyed. BEACH is a continuous, national cross-sectional study of general practice activity in Australia. Its methods are described in detail elsewhere [22]. In summary, an ever-changing, random sample of about 1,000 GPs per year each records information about encounters with 100 consecutive consenting patients, on structured paper forms [22].

In sub-studies of BEACH, the GP records information additional to the encounter data, in discussion with the patient. The full methods for sub-studies are reported elsewhere [22]. In this sub-study, 375 participating GPs were each asked to record diagnosed chronic conditions for each of 30 consecutive patients within their 100 BEACH records over three five week recording periods between 15^th^ July 2008 and 4^th^ May 2009.

**Figure 1:**
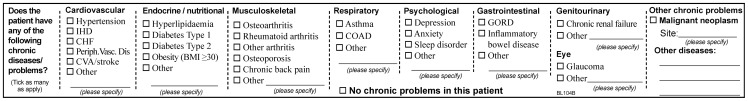
BEACH sub-study questionnaire on prevalence of chronic conditions.

Questions were brief, reducing the response burden on GPs and patients. GPs were asked, “Does the patient have any of the following chronic diseases/problems?” Common chronic conditions were listed (tick boxes) with additional blank spaces allowing free text descriptions of other unlisted chronic conditions (Figure 1). A “no chronic conditions” option was also provided. GPs were instructed to “Use your own knowledge, patient knowledge and health records as you see fit, in order to answer these questions”. Chronic conditions listed were primarily those most frequently managed among Australian general practice [22]. Other less frequently managed conditions (such as chronic kidney disease and obesity) were included where previous research had indicated they were prevalent in general practice patients [22]. All current National Health Priority area conditions were included [23]. Free text conditions were classified according to the International Classification of Primary Care (Version 2) (ICPC-2) [24].

### Data analysis

To ensure as many patients as possible were kept in the denominator, we examined GPs' response patterns for missing data. Where GPs ticked one or more conditions for some patients and did not tick any option (including “No chronic problems”) for other patients, the patients with no responses were compared with the total sample and the “No chronic problems” group. If patients with missing data resembled patients in the “No chronic problems in this patient” group in terms of age, sex and problems managed at encounter, we assumed the patients with no options ticked had none of the listed conditions, and they were counted as such. Patients with no options ticked but with any chronic condition (as defined by O'Halloran et al [25]) managed at encounter were also included in the sample, with the recorded chronic condition(s) counted in the sub-study.

BEACH sub-studies have a single-stage cluster design, with each GP having 30 patients clustered around them. The cluster effect was accounted for using SAS 9.2.

Sample prevalence estimates were the proportion of patients with the morbidity in the total sample and can be interpreted as prevalence among patients found in GP waiting rooms.

As patients were sampled at GP consultations, the likelihood of being sampled is dependent on visit frequency. Therefore frequent attenders (such as older patients who may have more health problems) were more likely to be sampled than infrequent attenders. Sample prevalence estimates were adjusted for this likelihood by weighting the sub-study sample against the age–sex distribution of the people who visited a GP at least once in 2008–09 (supplied by the Australian Government Department of Health and Ageing from Medicare claims data). We used 10 year age groups through to 90 years and over. Worked examples of all our weightings are in table 1. Applying these weights resulted in prevalence estimates for the general practice patient population (ie. those who saw a GP at least once that year).

**Table 1:** Worked examples of weighting method.

Formulas	Worked example: 80–89 year old female patient with condition X	Worked example: 10–19 year old male patient with condition X
A = Proportion of population that saw a GP at least once that year that was selected age–sex group	2.03%	5.83%
B = Proportion of the sample that was in the selected age–sex group	4.83%	3.03%
C = A/B (GP attenders weight)	0.42	1.92
D = Proportion of the total Australian population	1.87%	6.52%
E = D/B (National weight)	0.38	2.15
F = Number that saw a GP at least once that year (MBS GP item claims*)	362,815	1,040,270
G = Number in population (Australia Bureau of Statistics)	401,097	1,476,395
H = F/G (Proportion of age–sex group that saw a GP at least once that year)	90.46%	70.46%
Adjustment of outcome (or numerator) to estimate national prevalence = E*H	Condition X count = 0.34	Condition X count = 1.51
Denominator for national estimate (for both patients with and without condition) = E	0.38	2.15

* data supplied by the Australian Government Department of Health and Ageing.

To estimate national prevalence, we first weighted the sub-study sample against the age–sex distribution of the Australian population in June 2008–09 [26]. We assumed that people who did not attend a GP that year had no diagnosed chronic conditions. After the above weighting we multiplied the outcome (condition count) for each patient, by the proportion of their age-sex group who saw a GP at least once that year. This accounted for those who did not see a GP. This approach differs from our previous method where the general practice patient population prevalence was multiplied by the proportion of the whole population that attended at least once [18]. This new method will be more accurate if a higher proportion of older patients (than younger) attend at least once and if older patients are more likely to have a chronic condition.

We compared our national population prevalence with estimates from our previous paper [21] and from the NHS [7]. Significant differences with our earlier paper were determined by non-overlapping 95% confidence intervals (CIs). As CIs for the NHS [7] were not publicly available, we assumed that NHS estimates not within the 95% CIs of our population estimate were significantly different.

### Ethics statement

During the data collection period for this study the BEACH program was approved by the Human Research Ethics Committee of the University of Sydney and the Ethics Committee of the Australian Institute of Health and Welfare. Our method involves the collection of data from unidentifiable, consenting patients. A patient information card is supplied in the research kit, which GPs are instructed to show to patients in order to obtain informed consent (an example shown in Britt et al [22]). If the patient chooses not to participate their encounter details are not recorded. GPs are instructed to note the patient's consent in the patient's record, but are asked not to provide written consent to the research body, as this prevents patients remaining anonymous. These methods comply with the Ethics requirements for the BEACH program.

## Results

Completed research packs were returned by 290 GPs (77.3%) who responded for 8,333 (95.7%) patients out of a total 8,707. “No chronic problems in this patient” was ticked for 2,620 (31.4%) patients and 5,713 (68.6%) had at least one chronic condition recorded. Only 374 patients (4.3% of 8,707 patients sampled) had no response recorded. These were similar to patients with “No chronic problems”–with both groups being younger on average than the total sample and the majority of problems managed at their encounters were acute, whereas in the total sample these were mainly chronic problems. Sixty-four ‘no response’ patients had one or more chronic conditions managed at the encounter and were included as having these conditions while the remaining 310 ‘no response’ patients were added to the “No chronic problems” group. In total there were 8,707 patients in our sample with 5,777 (66.3%) having at least one chronic condition indicated and 2,930 (33.7%) with none.

The age-sex distribution of the final patient sample did not significantly differ from that of patients at all GP encounters claimed (as items of service) through Medicare in 2008–09 and was older than the population that attended a GP at least once that year (Table 2). The likelihood of at least one chronic condition increased significantly with patient age but did not differ among males and females.

**Table 2:** Age/sex distribution of sampled patients compared with all patients at GP service items claimed through Medicare and with the Australian general practice attending population.

Patient Age/Sex	Number in sample	Percent of sample (95% CIs)	Percent of Australian general practice service claims*	Percent of Australian general practice population†	Proportion of the sample with at least one chronic condition (95% CIs)
**Male**					
<15 years	595	6.9% (6.2–7.6)	7.3%	9.6%	19.8% (16.4–23.3)
15–24 years	272	3.2% (2.8–3.6)	3.3%	5.8%	32.7% (27.4–38.1)
25–44 years	735	8.5% (7.7–9.4)	8.6%	12.2%	56.3% (51.9–60.7)
45–64 years	1,020	11.8% (10.9–12.8)	11.8%	12.5%	82.1% (79.2–85.1)
65–74 years	487	5.7% (5.0–6.3)	5.8%	3.9%	96.1% (94.4–97.8)
75+ years	486	5.6% (5.0–6.3)	5.5%	2.8%	97.9% (96.7–99.2)
**Female**					
<15 years	565	6.6% (5.9–7.2)	6.5%	9.1%	16.8% (13.6–20.0)
15–24 years	497	5.8% (5.2–6.4)	6.0%	6.8%	39.4% (34.6–44.3)
25–44 years	1,297	15.1% (14.0–16.1)	14.5%	15.2%	52.1% (49.1–55.2)
45–64 years	1,405	16.3% (15.3–17.3)	15.6%	13.9%	81.0% (78.6–83.4)
65–74 years	550	6.4% (5.8–7.0)	6.7%	4.2%	94.2% (92.1–96.2)
75+ years	703	8.2% (7.1–9.2)	8.5%	4.1%	98.2% (97.1–99.2)

95 patients had either/both age or sex missing.

* Total MBS GP service items claimed during the 2008–09 BEACH year.

† Distribution of all patients that had at least one GP service item claimed in 2008–09.

### Sample prevalence

Cardiovascular problems were the most common, 31.3% having at least one, most prevalent being hypertension (26.6%) and ischaemic heart disease (8.7%) (Table 3). One or more endocrine/nutritional/metabolic diseases were present in 30.8% of patients, most commonly hyperlipidaemia (18.5%) and Type 2 diabetes (8.3%). Musculoskeletal conditions were present in 26.4% of patients, 19.7% having at least one type of arthritis (largely osteoarthritis 17.8%). One or more psychological problems were present in 22.1% of patients (13.7% depression and 8.3% anxiety). Asthma was indicated for 9.5% of patients and chronic obstructive airways/pulmonary disease (COAD/COPD) in 4.1%.

**Table 3:** Prevalence of selected chronic conditions in sample, attending population and Australian population.

Condition	Sample prevalence	Prevalence in those who attend at least once	Population prevalence	Knox et al. population estimates (2005) [21]	NHS estimates (2007) [7]
**At least one chronic condition**	66.3 (64.4–68.3)	58.8 (56.7–60.8)	49.6 (47.8–51.4)	46.8+ (45.0–48.5)	N/A
**Cardiovascular**	31.3 (29.4–33.1)	22.7 (21.2–24.2)	19.6 (18.3–20.9)	19.7 (18.4–21.0)	16.4
Hypertension	26.6 (24.9–28.4)	19.2 (17.8–20.6)	16.6 (15.4–17.8)	15.5 (14.4–16.6)	9.4
Ischaemic heart diseases	8.7 (7.7–9.8)	5.7 (5.0–6.4)	5.0 (4.4–5.6)	5.7 (5.0–6.3)	3.8^1^
Cerebrovascular accident	2.9 (2.3–3.5)	1.8 (1.4–2.1)	1.5 (1.2–1.8)	2.1 (1.7–2.6)	1.2^2^
Congestive heart failure	2.9 (2.4–3.4)	1.7 (1.4–2.1)	1.5 (1.2–1.8)	1.8 (1.5–2.1)	1.3^3^
**Endocrine, nutritional and metabolic diseases**	30.8 (29.0–32.6)	24.7 (23.2–26.3)	21.3 (19.9–22.6)	N/A	**
Hyperlipidaemia	18.5 (17.0–20.0)	14.1 (12.9–15.3)	12.3 (11.3–13.4)	11.2 (10.2–12.1)	5.7^4^
Diabetes mellitus	9.2 (8.3–10.1)	7.0 (6.3–7.7)	6.1 (5.5–6.7)	5.8 (5.3–6.4)	4.0
Type 1	0.9 (0.6–1.2)	0.8 (0.6–1.0)	0.7 (0.5–0.9)	0.5 (0.3–0.7)	0.4
Type 2	8.3 (7.5–9.1)	6.2 (5.6–6.9)	5.5 (4.9–6.0)	5.0 (4.5–5.5)	3.5
Obesity (BMI>30)	8.0 (7.0–8.9)	7.1 (6.2–7.9)	***	N/A	25.0^5^
**Musculoskeletal system and connective tissue**	26.4 (24.6–28.2)	19.6 (18.1–21.1)	16.7 (15.5–18.0)	N/A	30.7
Arthritis	19.7 (18.1–21.4)	13.8 (12.6–15.0)	11.9 (10.8–12.9)	14.8 (13.6–16.0)	15.2
Rheumatoid	1.0 (0.7–1.2)	0.7 (0.5–0.9)	0.6 (0.4–0.7)	0.7 (0.5–0.8)	2.1
Osteoarthritis	17.8 (16.2–19.4)	12.2 (11.0–13.3)	10.4 (9.4–11.4)	12.6 (11.5–13.7)	7.8
Other and unknown	2.0 (1.7–2.4)	1.6 (1.4–1.9)	1.5 (1.2–1.7)	N/A	6.1
Back pain	6.4 (5.5–7.2)	5.1 (4.4–5.8)	4.4 (3.8–5.0)	7.4 (6.5–8.2)	13.8^6^
Osteoporosis	4.8 (4.2–5.5)	3.0 (2.6–3.4)	2.4 (2.1–2.8)	N/A	3.4
**Psychological problems**	22.1 (20.6–23.7)	20.0 (18.5–21.5)	16.6 (15.3–17.8)	19.4 (18.1–20.8)	11.2
Depression	13.7 (12.6–14.7)	12.1 (11.1–13.1)	10.0 (9.2–10.8)	11.3 (10.3–12.4)	7.4^7^
Anxiety	8.3 (7.3–9.4)	7.6 (6.6–8.5)	6.2 (5.4–7.0)	8.4 (7.4–9.3)	3.3
Sleep disorder	3.0 (2.5–3.6)	2.6 (2.1–3.2)	2.2 (1.8–2.6)	N/A	N/A
Alcohol & drug problems	1.0 (0.6–1.4)	1.1 (0.7–1.5)	1.0 (0.6–1.3)	N/A	0.8
**Gastrointestinal**	14.6 (13.4–15.8)	11.3 (10.3–12.2)	9.6 (8.8–10.4)	N/A	**
GORD	11.6 (10.5–12.6)	8.8 (8.0–9.6)	7.5 (6.8–8.2)	9.2 (8.2–10.1)	N/A
**Respiratory disease**	13.7 (12.6–14.7)	12.5 (11.5–13.5)	10.5 (9.7–11.4)	N/A	**
Asthma	9.5 (8.7–10.3)	9.4 (8.6–10.3)	7.8 (7.1–8.5)	9.3 (8.5–10.2)	9.9
COAD/COPD	4.1 (3.4–4.7)	2.8 (2.3–3.3)	2.5 (2.1–2.9)	2.3 (1.9–2.6)	2.4^8^
**Malignant neoplasms**	5.0 (4.4–5.7)	3.6 (3.1–4.1)	3.1 (2.7–3.6)	2.0 (1.7–2.3)	1.6

N/A – Not available;

** Groups not comparable due to different inclusions;

*** Did not meet management assumption; GORD = gastro-oesophageal reflux disease; COAD/COPD = chronic obstructive airways disease/chronic obstructive pulmonary disease; +95% Confidence intervals were not reported in the earlier paper, they have been calculated for this paper;

NHS groups 1: Angina+other ischemic disease; 2: Cerebrovascular disease; 3: Odema+heart failure; 4: High cholesterol; 5: proportion of adults 18 years and older; 6: Back pain/problems, disc disorders; 7: Mood disorders; 8: Long term bronchitis+emphysema.

### General practice patient population

After adjustment, estimates for the general practice patient population were generally lower than sample estimates (Table 3) with 58.8% having at least one chronic condition. In particular, cardiovascular disease, arthritis and diabetes, (conditions common in older age), were significantly less prevalent after adjustment. Estimated prevalence of asthma and of psychological problems were largely unaffected by adjustment suggesting more similar prevalence of each across attending population age groups.

### Population prevalence

In 2008–09, 83% of the Australian population visited a GP at least once. After adjusting for non-attenders in each age-sex group, we estimated that 49.6% of the Australian population had at least one chronic condition, most commonly: endocrine problems (21.3%); cardiovascular problems (19.6%) and musculoskeletal problems (16.7%). Arthritis (any type) was present in 11.9%, asthma in 7.8% and gastro-oesophageal reflux disease (GORD) in 7.5% of the population. No estimate was made for obesity since it did not meet the assumption that it would not be present in non-attenders.

This study's estimate of the proportion of the population with at least one chronic condition was not significantly different to the 2005 study's estimate. For individual chronic problems there were few differences found between the two studies: our estimates for osteoarthritis, back pain and anxiety were significantly lower than our earlier study and the malignant neoplasm estimate significantly higher. Compared with the NHS, our population prevalence estimates were significantly higher for most cardiovascular conditions, hyperlipidaemia, osteoarthritis, diabetes mellitus, depression, anxiety and malignant neoplasms and significantly lower for rheumatoid arthritis, back pain, osteoporosis and asthma. There was agreement between the two estimates for congestive heart failure, COAD/COPD and alcohol and drug problems. No comparative results were available from NHS for GORD, sleep disorders, and the endocrine, gastrointestinal, and respiratory problem groups.

## Discussion

Despite differences in both the range of conditions surveyed and the data weighting methods, our prevalence estimates are consistent with our earlier study [21]. This study has shown that nearly two-thirds of patients sitting in front of the GP and half of the Australian population had at least one chronic condition. These sample prevalence estimates provide a measure of underlying health needs of patients attending general practice, distinct from demand for health care measured by general practice morbidity management rates. However, not surprisingly, the most prevalent problems in our sample were similar to those most often managed in general practice [22].

Inclusion criteria may explain some of the differences between NHS estimates and our estimates. For example, our definition of “back pain” was limited to chronic back pain whereas the NHS, included all types of back issues. Another possible cause for differences is the NHS's reliance on respondent self-report, e.g. confusion between terms “arthritis” and “rheumatism” may explain why the NHS produced a far higher estimate of the prevalence of “rheumatoid arthritis”.

While our prevalence estimate of psychological problems (16.6%) was about 50% higher than the NHS estimate it was closer to the 2007 National Mental Health and Wellbeing Survey estimate, that one-in-five Australians had experienced a psychological problem during the previous year [27]. Our prevalence estimate for hypertension (16.6%) lay between that of the NHS (9.4%) and 2005 AusDiab [28](31.1%) estimates. However, one would expect AusDiab's result to be higher for two reasons. Firstly, they measured blood pressure only once as per WHO guidelines for field testing [29] whereas a GP will use repeated measures before diagnosis [30]. Secondly, they included patients whose blood pressure was normal, but were taking antihypertensives. This would have included those without diagnosed hypertension prescribed antihypertensives to lower their cardiovascular risk from another condition such as diabetes [30].

The largest difference in estimates was for obesity. Our study suggested that only 8.0% of patients sitting in front of the GP are obese. This is far lower estimate than the 25.0% of adult patients found in the NHS [7] and 26.7% in other large BEACH sub-studies where patients self-report height and weight [22]. Many may find the low prevalence found in our study of concern, especially when one considers that obesity is infrequently managed in general practice as a condition in its own right [22]. However, while obesity is not frequently managed as an identified condition, in the management of other problems counselling about diet and exercise is one of the most frequent treatments given by GPs in Australian general practice [22]. When obesity is managed in general practice, the majority of the time the patient has raised it as an issue they want managed [31]. This suggests that patients' desire for treatment plays a strong role in whether a GP manages obesity as a condition in its own right. Our prevalence estimate of 8.0% does however match the 8.1% of patients with morbid obesity (BMI of 35+) found in previous research [32]. This may suggest that GPs in our study are identifying patients who have a more extreme “chronic” level of obesity.

Our slightly higher prevalence estimate of at least one chronic condition compared with our previous study is probably due to our inclusion of all chronic conditions rather than only a selection. However, the ageing population or increases in diagnoses could also have contributed to this difference.

Our study has limitations. We assumed that people who did not see their GP in the previous year did not currently have a diagnosed chronic condition. This assumption may not hold for conditions such as asthma, where it is mild and did not necessitate a GP attendance that year. This may explain our lower prevalence estimate for asthma compared with NHS.

An issue with measuring diagnosed chronic conditions is that, like most prevalence studies, we can only provide estimates for those conditions already diagnosed. As the Ausdiab study shows, a significant proportion of Australians have undiagnosed diabetes and hypertension [28].

Finally our sample was drawn from patients attending general practice, so we were more likely to sample people who attend more frequently. While we adjusted for higher attendance of female and older patients, our method could not adjust for high attenders within a specific ten year age-sex group. If patients with particular conditions consistently attend more often than the average for their age and sex, this could lead us to overestimate prevalence of these conditions.

## Conclusion

This study provides the only current prevalence data that uses the GP as an expert interviewer and informant to gather information from the patient, their knowledge of the patient, and the health record. For a marginal cost to the BEACH program, this investigation could be run on an annual basis and could be expanded to 30,000 patients per year if larger samples were required. Our estimates can be used to examine primary care resource use in management of these chronic conditions. Importantly, the increased scope of this study allows measurement of prevalence of all chronic conditions and can therefore be used to measure prevalence of multimorbidity in Australia. To further increase the accuracy of estimates, the next version of this study will include a question on the number of patient visits to any GP in the past year so we can adjust for intra age-sex group variation in visit frequency.
